# Tonabersat Inhibits Connexin43 Hemichannel Opening and Inflammasome Activation in an In Vitro Retinal Epithelial Cell Model of Diabetic Retinopathy

**DOI:** 10.3390/ijms22010298

**Published:** 2020-12-30

**Authors:** Heather Lyon, Avik Shome, Ilva D. Rupenthal, Colin R. Green, Odunayo O. Mugisho

**Affiliations:** Buchanan Ocular Therapeutics Unit, Department of Ophthalmology, New Zealand National Eye Centre, Faculty of Medical and Health Sciences, University of Auckland, Auckland 1023, New Zealand; heather-lyon@hotmail.co.uk (H.L.); a.shome@auckland.ac.nz (A.S.); i.rupenthal@auckland.ac.nz (I.D.R.); c.green@auckland.ac.nz (C.R.G.)

**Keywords:** connexin43, hemichannels, inflammation, inflammasome, diabetic retinopathy, tonabersat

## Abstract

This study was undertaken to evaluate the connexin hemichannel blocker tonabersat for the inhibition of inflammasome activation and use as a potential treatment for diabetic retinopathy. Human retinal pigment epithelial cells (ARPE-19) were stimulated with hyperglycemia and the inflammatory cytokines IL-1β and TNFα in order to mimic diabetic retinopathy molecular signs in vitro. Immunohistochemistry was used to evaluate the effect of tonabersat treatment on NLRP3, NLRP1, and cleaved caspase-1 expression and distribution. A Luminex cytokine release assay was performed to determine whether tonabersat affected proinflammatory cytokine release. NLRP1 was not activated in ARPE-19 cells, and IL-18 was not produced under disease conditions. However, NLRP3 and cleaved caspase-1 complex formation increased with hyperglycemia and cytokine challenge but was inhibited by tonabersat treatment. It also prevented the release of proinflammatory cytokines IL-1β, VEGF, and IL-6. Tonabersat therefore has the potential to reduce inflammasome-mediated inflammation in diabetic retinopathy.

## 1. Introduction

Diabetic retinopathy (DR) is a chronic retinal disease resulting from hyperglycemia and inflammation-linked vascular pathology. It is characterized by blood–retinal barrier (BRB) leakage and neovascularization in late stages, leading to vision loss [1,2,3]. Currently, the gold-standard treatment for DR consists of anti-vascular endothelial growth factor (anti-VEGF) drugs [4,5]. However, anti-VEGF drugs have several drawbacks, including the potential for increased geographic atrophy from long-term use, resistance to treatment in some patients, failure to treat the early stages of DR, and a lack of specificity to address the underlying cause [6,7,8,9,10]. As such, alternative treatment avenues are actively being sought.

One option being explored involves targeting upstream inflammation pathways such as the NOD-like receptor protein 3 (NLRP3) inflammasome pathway. A priming signal induces the translocation of nuclear factor kappaB (NF-κB) to the nucleus as part of the NLRP3 inflammasome pathway immune response. NF-κB induces the expression of various proinflammatory genes, including those encoding cytokines and chemokines. In a second step, NLRP3 inflammasome activation triggers cleavage, activation, and secretion of proinflammatory IL-1β and IL-18, which then recruit different types of effector cells and coordinate the innate immune response.

Studies have shown that NLRP3 inflammasome activation seen in DR is associated with the opening of connexin43 hemichannels under conditions associated with chronic disease [11,12]. Connexin43 is the most common connexin isotype in the human body, forming gap junctions (GJ) when two hemichannels, each composed of six connexins, dock between neighboring cells and allow the shared passage of ions, signaling molecules, and metabolites [13,14,15,16,17].

However, connexin43 hemichannels can exist undocked in cell membranes, with recent research suggesting that these mediate deleterious effects correlated with increased connexin43 expression [11,18,19]. Connexin43 hemichannel opening has been shown to activate the inflammasome pathway by mediating an ATP autocrine feedback loop, both allowing an initial release of ATP triggering the assembly of the NLRP3 inflammasome and continued release, amplifying and perpetuating the inflammasome pathway [11]. In that study, blocking connexin43 hemichannels with Peptide5 prevented ATP release, inhibited NLRP3 inflammasome activation, and reduced downstream proinflammatory cytokine release. Thus, connexin43 hemichannel blockers offer a promising option for the treatment of inflammasome-pathway-associated diseases, such as DR, with the possibility of treating patients more successfully and in earlier stages of the disease [20].

Several previous studies establishing the mechanism by which connexin43 hemichannels regulate the inflammasome have used Peptide5 as a hemichannel blocking agent, a mimetic peptide derived from the second extracellular loop of the connexin43 protein. Given that this drug is a peptide, its delivery method for ocular disease is more limited, for example, to intravitreal injection. Another connexin43 hemichannel blocker, tonabersat, is being studied as an alternative to Peptide5. Tonabersat is a small-molecule benzopyran derivative that crosses the blood–brain barrier and has been shown to block connexin43 hemichannels to prevent signs of ocular pathology in several in vivo disease models following systemic and oral delivery [21,22]. However, while tonabersat has been shown to act as a connexin43 hemichannel blocker [21], its complete mode of action in disease conditions is yet to be proven.

Therefore, the aim of the present study is to evaluate the means by which tonabersat acts to prevent molecular markers of DR using human retinal pigment epithelial cells (ARPE-19) stimulated with hyperglycemia and inflammatory cytokines (HG + Cyt) to mimic molecular DR markers in vitro (Kuo et al., 2020). Given that a mode of action has already been described using Peptide5, we looked to determine if tonabersat acts similarly to Peptide5, i.e., by blocking ATP release and, ultimately, inflammasome activation. In the first instance, ATP release profiles, as well as cell viability, were assessed following tonabersat treatment and compared to Peptide5 treatment. Subsequently, immunohistochemistry was used to evaluate the effect of tonabersat treatment on NLRP3 and cleaved caspase-1 expression and distribution. A Luminex cytokine release assay was performed to determine whether tonabersat affects proinflammatory cytokine release from cells. Immunohistochemical analysis of inflammasome activation, as well as cytokine release measurements, have previously been conducted using Peptide5 [11]. This study showed that hemichannel block with Peptide5 reduced ATP release after the insult back to baseline levels, subsequently stopped inflammasome complex assembly, shown by immunohistochemistry speckle labeling, and significantly reduced the release of IL-6, sICAM-1, MCP-1, IL-8, and VEGF. The addition of exogenous ATP negated the Peptide5 treatment effect, confirming it was specifically ATP release that was activating inflammasome complex assembly. Finally, in this current study, we have investigated the effect of HG + Cyt with and without tonabersat on another inflammasome-forming NLR protein, NLRP1, to ascertain whether the mode of action in DR is primarily via the NLRP3 inflammasome pathway.

## 2. Results

### 2.1. Tonabersat Prevented HG + Cyt-Induced ATP Release in a Similar Manner to Peptide5

**Figure 1:**
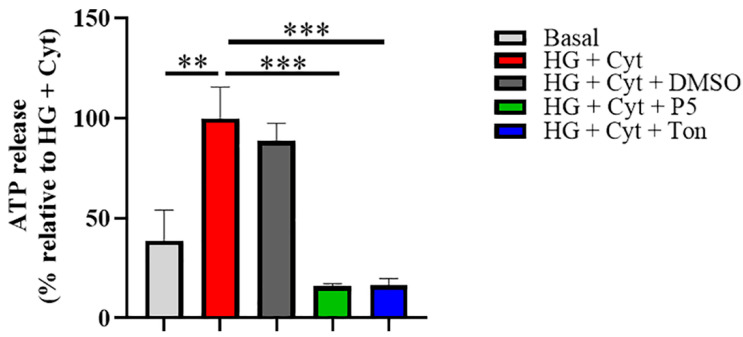
Effect of tonabersat and Peptide5 treatment on hyperglycemia and inflammatory cytokines (HG + Cyt)-induced ATP release. HG + Cyt induced a significant increase in ATP release compared to the untreated group (*p* = 0.0053). Both tonabersat (HG + Cyt + Ton; *p* = 0.0009) and Peptide5 (HG + Cyt + P5; *p* = 0.0003) treatment significantly decreased ATP release to below basal levels (not significantly different). DMSO treatment (HG + Cyt + DMSO) did not induce or inhibit ATP levels. Data are presented as mean + SEM. Statistical analysis was carried out using one-way ANOVA with Dunnett’s multiple comparisons test. ** *p* ≤ 0.01; *** *p* ≤ 0.001, *n* = 3.

The results showed that HG + Cyt challenge (100.0 + 15.6%) increased ATP levels released into the media relative to the untreated group (38.9 + 15.3%, *p* = 0.0053) (Figure 1). DMSO alone (88.5 + 8.8%) had no effect on ATP release relative to HG + Cyt, but both tonabersat (16.4 + 3.3%, *p* = 0.0009) and Peptide5 (15.9 + 1.3%, *p* = 0.0003) treatments blocked ATP release with extracellular levels below those of untreated cells. There was no statistically significant difference in ATP release between tonabersat and Peptide5 treatment and the untreated cell (normal, unchallenged cells) levels. There was no difference between tonabersat and Peptide5 effects on HG + Cyt challenged cells, indicating that they have equivalent ability to block ATP release at the concentrations used.

### 2.2. Tonabersat Treatment Inhibited HG + Cyt-Induced Proinflammatory Cytokine Release

**Figure 2:**
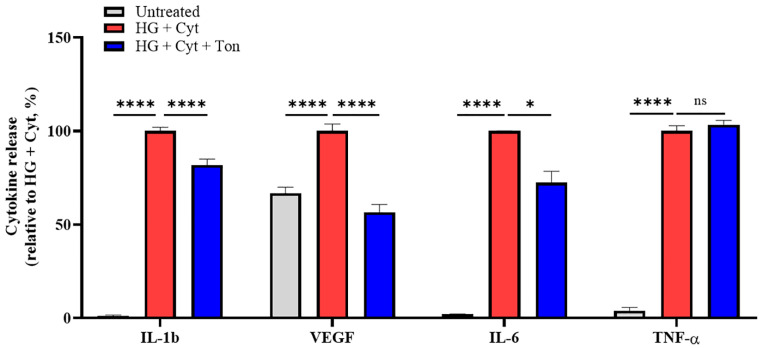
Effect of tonabersat treatment on HG + Cyt-induced proinflammatory cytokine release. All four cytokines, IL-1β, VEGF, IL-6, and TNF-α, were significantly increased following HG + Cyt treatment in comparison to untreated conditions at 24 h (there was no IL-1β prior to the challenge). Tonabersat treatment (HG + Cyt + Ton) significantly reduced IL-1β, VEGF, and IL-6 but not TNF-α levels relative to the HG + Cyt group. Data are presented as mean + SEM. Statistical analysis was carried out using one-way ANOVA with Dunnett’s multiple comparisons test. ns *p* > 0.05; * *p* < 0.05; **** *p* ≤ 0.0001; *n* = 3.

We previously discovered that blocking connexin hemichannels with Peptide5 can inhibit proinflammatory cytokine release. Here, we examined the effect of tonabersat on the release of the proinflammatory cytokines IL-1β, TNF-α, IL-6, and VEGF at 24 h after the commencement of the various treatments (Figure 2). The results showed that: (1) HG + Cyt (IL-1β = 100.0 + 2.0%; VEGF = 100.0 + 3.7%; IL-6 = 100.0 + 0.1%; and TNF-α = 100.0 + 2.9%) significantly increased the release of all three cytokines relative to the untreated cells (IL-1β = 1.2 + 0.4%; VEGF = 66.7 + 3.3%; IL-6 = 2.1 + 0.1%; TNF-α = 4.0 + 1.7%; all *p* < 0.0001); (2) tonabersat treatment (HG + Cyt + Ton) led to a significant reduction in IL-1β (81.9 + 3.2%; *p* < 0.0001), VEGF (56.5 + 4.1%, *p* < 0.0001) and IL-6 (72.3 + 6.1, *p* = 0.0104) levels, but had no effect on TNF-α levels (103.4 + 2.2%, *p* = 0.5203) levels compared to HG + Cyt alone. IL-18 was measured but not produced by these cells (data not shown).

### 2.3. Tonabersat Treatment Prevented NLRP3 Inflammasome Assembly in an ATP-Dependent Manner

**Figure 3:**
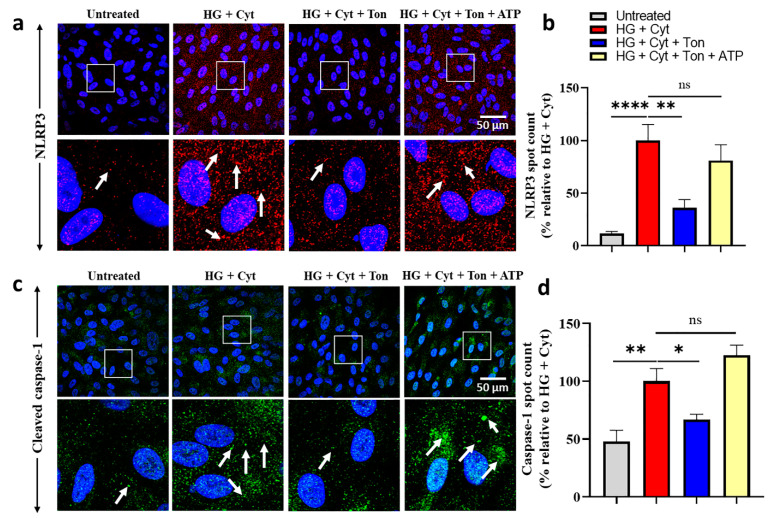
Effect of tonabersat treatment and exogenous ATP addition on HG + Cyt-induced NLRP3 protein and cleaved caspase-1 assembly into inflammasomes. Panel (**a**) shows immunohistochemistry speckle labeling of NLRP3 incorporated into inflammasome complexes, whilst (**b**) shows spot count quantification of that data. Tonabersat treatment (HG + Cyt + Ton) prevented HG + Cyt-induced NLRP3 complex assembly. NLRP3 spot count data showed that HG + Cyt significantly increased NLRP3 spot counts relative to untreated conditions. In comparison, tonabersat treatment significantly prevented an increase in the NLRP3 complex assembly, which remained at baseline levels. The addition of exogenous ATP reversed the protection conferred by tonabersat with no significant difference between the ATP and injury alone groups, indicating that connexin hemichannel-mediated ATP release was the complex assembly-activating signal, with hemichannel block overridden by the addition of exogenous ATP. Panel (**c**) shows immunohistochemical speckle labeling of caspase-1 incorporated into inflammasome complexes, whilst (**d**) shows spot count quantification of that data. Tonabersat treatment also prevented an HG + Cyt-induced increase in cleaved caspase-1 incorporation into inflammasome complexes. HG + Cyt significantly increased cleaved caspase-1 spot counts relative to the untreated group. Tonabersat treatment significantly inhibited HG + Cyt-induced cleaved caspase-1 upregulation, and again, exogenous ATP addition reversed tonabersat effects with no significant difference between the ATP and HG + Cyt groups, indicating that connexin hemichannel-mediated ATP release was the complex assembly-activating signal, with hemichannel block overridden by the addition of exogenous ATP. Boxed areas (50 µm square) are enlarged; arrows illustrate labeled NLRP3 or cleaved caspase-1 spots. Data are presented as mean + SEM. Statistical analysis was carried out using one-way ANOVA with Dunnett’s multiple comparisons test. ns *p* > 0.05; * *p* < 0.05; ** *p* ≤ 0.01; **** *p* ≤ 0.0001; *n* = 5; scale bar = 50 µm.

When inflammasome complexes assemble, multiple copies of NLRP3 are incorporated and complex assembly can be assessed using NLRP3 immunohistochemistry. We have previously shown that hemichannel blocker treatment with Peptide5 prevented NLRP3 aggregation [11]. In this study, we assessed the effect of tonabersat treatment on NLRP3 expression and localization. The NLRP3 inflammasome complex assembly (visualized as speckle label) was visibly different across treatment groups (Figure 3a) and confirmed through spot count quantification (Figure 3b). HG + Cyt challenge led to a significantly higher NLRP3 spot count (100.0 + 15.2%) than the untreated group (11.7 + 1.9%; *p* < 0.0001), and NLRP3 assembly was significantly reduced with tonabersat treatment (36.0 + 7.9%; *p* = 0.0022). Coapplication of exogenous ATP with tonabersat, however, reversed any protective effects, with no significant difference found between the HG + Cyt and HG + Cyt + Ton + ATP (81.0 + 15.1%; *p* = 0.4971) groups, supporting the idea that connexin hemichannel-mediated ATP release was the activating signal, with its block overridden by the addition of exogenous ATP.

### 2.4. Tonabersat Treatment Prevented Upregulation of Cleaved Caspase-1 in an ATP-Dependent Manner

The inflammasome mediates the activation of caspase-1, which in turn cleaves inflammatory cytokines IL-1β and IL-18 for release from the cell. Similar to NLRP3, cleaved (activated) caspase-1 aggregates within inflammasome complexes and can be visualized by immunohistochemistry and quantified by a spot count. The spot count was significantly higher following HG + Cyt challenge (100.0 + 11.0%) than in the untreated conditions (47.9 + 9.7%; *p* = 0.0020) (Figure 3c,d). Tonabersat treatment (HG + Cyt + Ton; 66.7 + 4.7%) resulted in a significant reduction in cleaved caspase-1 spot counts in comparison to HG + Cyt (*p* = 0.0433). Coapplication of exogenous ATP reversed this protection (122.6 + 8.7%; *p* = 0.2066) (Figure 3d), again indicating that connexin hemichannel-mediated ATP release was the activating signal, with its block overridden by the addition of exogenous ATP.

### 2.5. Neither HG + Cyt Challenge Nor Tonabersat Treatment Affected NLRP1 Levels or Localization

**Figure 4:**
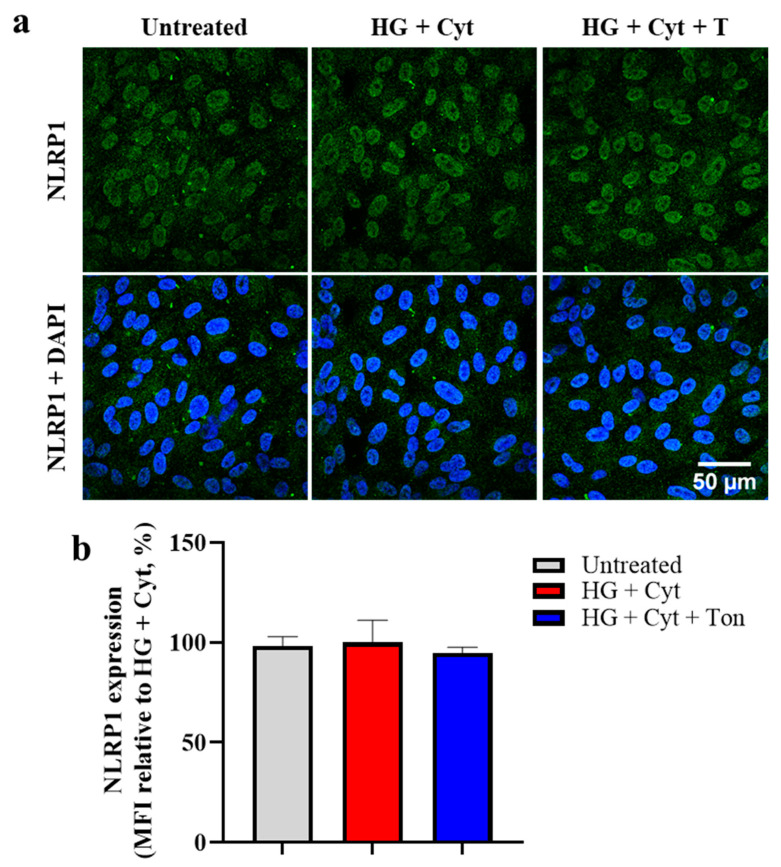
Effect of HG + Cyt and connexin43 hemichannel block with tonabersat on NLRP1 labeling intensity or distribution. Panel (**a**) shows NLRP1 localization remained consistently nuclear across all treatment groups. No changes in NLRP1 protein localization (**a**) or total labeling levels (mean fluorescence intensity) (**b**) were observed following HG + Cyt challenge or the addition of tonabersat, indicating that NLRP1 is not activated in this model. Data are presented as mean + SEM. Statistical analysis was carried out using one-way ANOVA. *n* = 5; scale bar = 50 μm.

NLRP1 is another inflammasome-forming protein, with its involvement in DR-like conditions currently unclear. The results showed mild nuclear NLRP1 labeling consistent across all treatment groups. Furthermore, neither NLRP1 labeling intensity nor localization was significantly affected by any of the treatment conditions (Figure 4a,b), suggesting that the NLRP1 inflammasome is not activated under these challenge conditions in this model.

## 3. Discussion

Recent studies have connected a heightened connexin43 hemichannel opening probability under injury conditions with the pathology of various retinal diseases (Danesh-Meyer et al., 2016b; Mugisho et al., 2019). Within the retina, one such disease is DR, whereby BRB leakage and neovascularization can lead to vision loss. Current DR treatments are anti-VEGF drugs that target the neovascularization arm of the DR pathology. However, these fail to treat either the early stages of DR or the root cause of the disease (Campochiaro et al., 2016; Dhoot and Avery, 2016). As such, connexin43 hemichannel blockers offer a promising alternative, directly targeting a fundamental upstream pathway in the disease pathogenesis, and one that may result in perpetuating the disease once initiated [11]. Previous studies have explored the efficacy of connexin43 hemichannel blockers for the treatment of DR in both in vitro and in vivo models [19,20,22]. These studies have shown that connexin43 hemichannel block with Peptide5 or tonabersat prevented both inflammatory and vascular signs of the disease. Tonabersat has also been shown to block connexin43 hemichannels and in an age-related macular degeneration model, where it was found to inhibit loss of electroretinogram function as well as retinal cell death and inflammation [21,22].

In a bid to better understand the mode of action by which connexin43 hemichannel block protects against cell death and inflammation, we previously evaluated the effect of Peptide5 on an innate immune system pathway, the NLRP3 inflammasome [11]. Molecular and cellular DR pathology was induced using a combination of HG and the proinflammatory cytokines, IL-1β and TNF-α, a combination that is known to result in the opening of connexin43 hemichannels [11,12,19]. It was demonstrated that connexin43 hemichannel block with Peptide5 inhibits pathological ATP release, which in turn prevents the aggregation of NLRP3 inflammasome complexes and downstream autolytic cleavage and activation of caspase 1.

Activation of caspase-1 is required to cleave the proforms of both IL-1β and IL-18 for release from the cell (for a pathway diagram see [11]). Mugisho and colleagues showed that the protection conferred by hemichannel block with Peptide5 could be reversed by the addition of exogenous ATP, suggesting that ATP alone could be sufficient for activation of the inflammasome pathway. While these results are important to our understanding of the role of connexin43 hemichannels in DR, they were conducted using a single hemichannel blocker, Peptide5. The aim here was to determine the mode of action of another hemichannel blocker, tonabersat, an orally available, Phase-2-ready small molecule, by investigating whether it modulates activation of the inflammasome pathway in a manner similar to the well-characterized hemichannel blocker Peptide5.

Our initial results showed that tonabersat inhibited HG + Cyt-induced ATP release to a similar extent as Peptide5 and as previously published [11]. Furthermore, DMSO at the concentration used in the tonabersat group did not affect ATP release, suggesting that any effects attributed to the tonabersat group are independent of the solubilizing vehicle. Next, we studied the localization of the inflammasome markers NLRP3 and cleaved caspase-1. Immunohistochemistry results demonstrated that HG + Cyt challenge increased NLRP3 complex assembly, as shown by the increase in punctate NLRP3 and cleavage of caspase-1 and as shown by increased cleaved caspase-1 labeling with an antibody that is specific for the cleaved form of the protein. Tonabersat treatment prevented NLRP3 complexation, similar to the previously reported Peptide5 effects [11] and reduced cleavage of caspase-1 into its active form. We also evaluated the effect of HG + Cyt challenge and connexin43 hemichannel block on NLRP1, the other NLR thought to also play a crucial role in inflammasome activation in retinal degenerative diseases [23]. In the eye, NLRP1 has been implicated in both DR and glaucoma with studies suggesting that upregulation of NLRP1 contributes to inflammation, ganglion cell death, and neovascularization [24,25,26,27,28]. Our results showed that NLRP1 was not affected by any of our treatments, suggesting that the inflammasome complex assembly triggered by HG + Cyt is independent of the NLRP1 inflammasome. Whilst these findings appear to contradict previous studies that support the role of the NLRP1 inflammasome in DR, that work was conducted in endothelial cells or mouse models with all retinal cells present. To the best of our knowledge, no studies have evaluated NLRP1 roles using RPE cells specifically, and it is probable that NLRP1 may not be activated and/or activated in RPE cells. It is also worth noting that NLRP1 roles in disease remain highly debated due to its apparent dichotomous effect in disease [24]. Whilst the present study suggests that NLRP3 is instrumental in DR-mediated RPE damage, more studies are required to fully elucidate NLRP1 inflammasome involvement in retinal diseases.

The effect of tonabersat treatment on the inflammasome was also reflected in the proinflammatory cytokine release profile observed. Tonabersat treatment significantly decreased the secretion of IL-1β, a major marker for inflammasome activation. It is important to note that IL-1β released was not restored to basal levels, possibly due to the fact that exogenous IL-1β was one of the two cytokines added to the culture medium to induce injury in the first instance. This is a limitation of the study because it is difficult to differentiate between exogenously added and induced IL-1β. The other cytokines, VEGF, IL-6, and TNF-α, were all significantly increased by HG + Cyt relative to the untreated group, with both VEGF and IL-6 being significantly reduced by tonabersat treatment. The release of IL-1β is assumed to induce the secretion of other cytokines since it is known that IL-1β induces both VEGF and IL-6 transcription in other cell types [29,30,31]. However, TNF-α secretion was not reduced by hemichannel block, suggesting that an NLRP3 inflammasome-independent TNF-α secretion pathway had been activated [32]. In that article, it was reported that deficiency of neither NLRP3 nor caspase-1 had an effect on the secretion of TNF-α. It is also of note that TNF-α induces TNF-α promoter activation in an autocrine manner [33]. This supports the argument that while the tonabersat mode of action in blocking the connexin hemichannel leads to specific actions on the NLRP3 inflammasome, hemichannel block with tonabersat is not acting on inflammasome-independent pathways.

**Figure 5:**
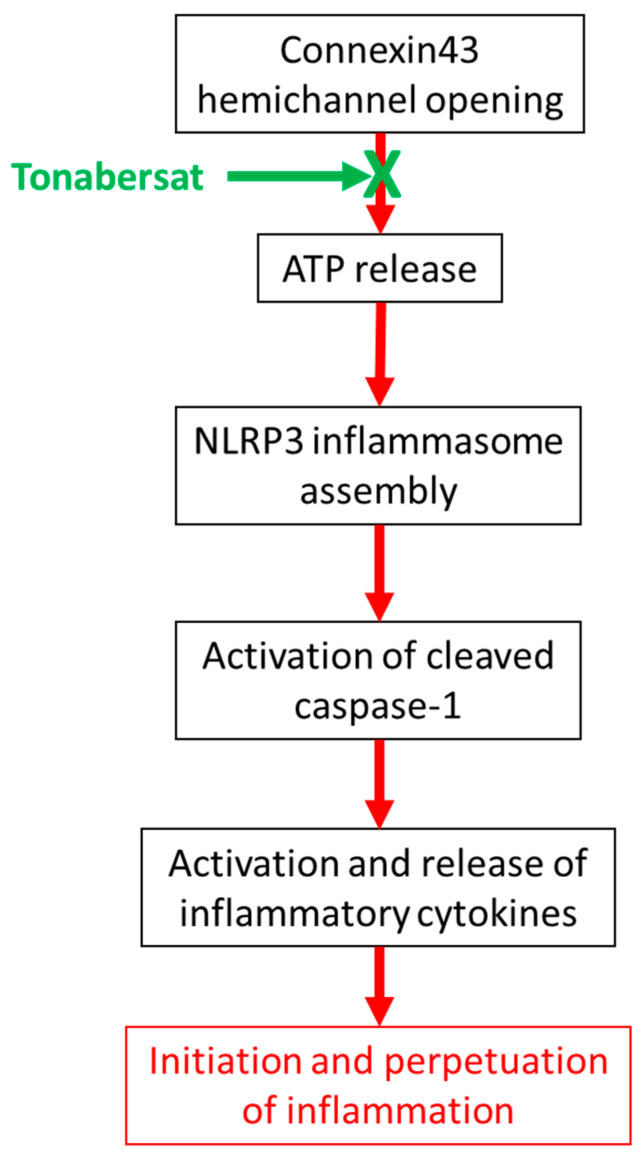
A schematic diagram outlining the mode of action of tonabersat. Connexin hemichannel-mediated ATP release triggers inflammasome complex assembly. Block of the connexin43 hemichannel with tonabersat prevents NLRP3 and cleaved caspase-1 complex formation and the release of inflammatory cytokines.

In conclusion, the present study demonstrated that tonabersat treatment inhibited ATP-mediated inflammasome activation in the same manner as another well-characterized hemichannel blocker, Peptide5 (Figure 5). Tonabersat treatment prevented NLRP3 and cleaved caspase-1 complex assembly, as well as release of the proinflammatory cytokines IL-1β, VEGF, and IL-6. The NLRP1 inflammasome was not activated in this study, suggesting that NLRP3 is the key inflammasome activated within the RPE in DR. Tonabersat has the potential to reduce inflammasome-mediated inflammation in DR.

## 4. Materials and Methods

### 4.1. Cell Culture

Human adult retinal pigment epithelial cells (ARPE-19; American Type Culture Collection, Manassas, USA) were cultured in Dulbecco’s modified Eagle medium F-12 (DMEM-F12; Thermofisher Scientific New Zealand Ltd., Auckland, New Zealand) supplemented with 10% fetal bovine serum (FBS; Invitrogen, Carlsbad, USA) and a 1× antibiotics and antimycotics mixture (AA, 100× stock). ARPE-19 cells were grown in T75 flasks in humidified 5% CO_2_ at 37 °C and subcultured every seven days, with the medium changed twice per week. Immunohistochemistry was used to confirm the RPE phenotype in the cultured ARPE-19 cell line using antibodies against retinal pigment epithelium-65 (RPE-65) (Supplementary Figure S1a).

### 4.2. High Glucose and Cytokine Challenge and Application of Treatments

Cells of passage 7–14 were plated at 2.5 × 105 cells/mL in 8-well chamber slides for immunohistochemical studies, 96-well plates for cell viability, or 24-well plates for the Luminex cytokine release assays. Cells were treated once confluency over 90% was achieved. There were four treatment groups: untreated, high glucose and cytokines (HG + Cyt), HG + Cyt plus tonabersat (HG + Cyt + Ton), and HG + Cyt + Ton plus 20 nM ATP (HG + Cyt + Ton + ATP). For initial experiments where tonabersat was compared to Peptide5, an additional group of HG + Cyt plus Peptide5 (HG + Cyt + P5) was included. The HG + Cyt challenge was a combination of 32.5 mM HG and the proinflammatory cytokines interleukin-1 beta (IL-1β; 10 ng/mL; Peprotech, Rocky Hill, USA) and tumor necrosis factor-alpha (TNF-α; 10 ng/mL; Peprotech, Rocky Hill, USA) to induce DR-like conditions as previously described (Kuo et al., 2020; Mugisho et al., 2018a, 2018b). Experiments assessing the effects of exogenous ATP (20 nM; Sigma-Aldrich, St Louis, USA) were conducted as previously described [11,19]. Briefly, exogenous ATP was added to the cell culture media at the same time as other treatments.

Connexin43 hemichannel blockers tonabersat (MedChemExpress, Monmouth Junction, USA) and Peptide5 (H-Val-Asp-Cys-Phe-Leu-Ser-Arg-Pro-Thr-Glu-Lys-Thr-OH; China Peptides, Shanghai, China) were administered at 100 µM and 20 µM, respectively, to cells along with HG + Cyt. Tonabersat was dissolved in 100% dimethyl sulfoxide (DMSO) at a concentration of 100 mM, after which 1 µL of the stock solution was added to 999 µL of culture medium containing HG + Cyt. To confirm that the DMSO solution did not produce any cytotoxic effects, a DMSO treatment group (HG + Cyt + DMSO) was added (Supplementary Figure S1b). Peptide5 was reconstituted in phosphate-buffered saline (PBS). Following treatment, cells were incubated under treatment conditions for 24 h unless otherwise stated. Brightfield images were taken using a light microscope at 24 h post-treatment. Each experiment was conducted in triplicate, and experiments repeated three times to confirm results were consistent.

### 4.3. ATP Release Assay

To measure ATP release following treatment, ATP assays were carried out on the cell culture supernatant after 24 h in culture. Cell culture media samples were collected in duplicates from a total of three wells per condition. ATP release was measured using the ATPLite Luminescence ATP Detection Assay System (PerkinElmer, Waltham, MA, USA), in line with the manufacturer’s instructions. ATP release (%) was calculated relative to cells treated with HG + Cyt (DR-like challenge group). ATP release was measured on three independent occasions.

### 4.4. Cytokine Profiling Using the Luminex Bead Array

To determine the effect of treatment conditions on proinflammatory cytokine release, a human Magnetic Luminex assay was performed in accordance with manufacturer guidelines (R&D Systems, Minneapolis, MN, USA). The analytes targeted were IL-1β, VEGF, IL-6, TNF-α, and IL-18. The supernatant was sampled at 24 h post-treatment, from three separate wells per condition. Standard cocktails were used to determine the concentration levels of each target analyte. Samples were read using a Luminex MAGPIX reader (R&D Systems, Minneapolis, MN, USA). Cytokine levels in treated cells were presented relative to their respective HG + Cyt challenge group.

### 4.5. Immunocytochemistry

Following treatment, cells were fixed with 4% paraformaldehyde for 10 min before permeabilization with 0.1% Triton X-100 in PBS for 10 min. Cells were then blocked for 1 h in normal goat or horse serum before overnight incubation at 4 °C with their respective primary antibodies; anti-NLRP3 (1:500; Abcam, Cambridge, UK), anti-cleaved caspase-1 (1:100, Invitrogen, Carlsbad, USA), anti-NLRP1 (1:500; Novus Biologicals, Littleton, CO, USA), or mouse anti-RPE65 (1:1000; Abcam, Cambridge, UK). Two 10 min washes in PBS followed, after which cells were incubated at room temperature for 2 h with their respective secondary antibodies; donkey anti-rabbit Alexa-488 (1:500; Abcam, UK), donkey anti-mouse Alexa-488 (1:500; Abcam, Cambridge, UK), or donkey anti-goat Cy3 (1:500; Invitrogen, Carlsbad, USA). Cells were then washed twice in PBS for 10 min. Cell nuclei were counterstained with DAPI (1:1000; Sigma-Aldrich, St Louis, MO, USA), and slides were mounted using a CitifluorTM antifade reagent.

### 4.6. Image Analysis and Antibody Quantification

An Olympus FV1000 confocal laser scanning microscope (Olympus, Tokyo, Japan) was used to take immunofluorescence images, which were then processed using Olympus FV10-ASW software and version 1.52a ImageJ software (National Institute of Health, Bethesda, MD, USA). Five images were taken per chamber. The images were quantified following image conversion to binary 8-bit and equal thresholding to reduce background interference. NLRP3 and cleaved caspase-1 expression was determined by counting protein aggregates (assembled inflammasome complexes) shown as punctate spots. NLRP1 expression was measured by quantifying the mean fluorescence intensity (MFI) of the entire image.

### 4.7. Statistical Analysis

Data are presented as arithmetic mean + SEM. All statistical comparisons were made using GraphPad Prism 8 (GraphPad Software, San Diego, CA, USA) A one-way ANOVA was commonly performed, followed by Dunnett’s post-hoc testing, to compare the HG + Cyt challenge to other conditions. The specific statistical method used for each data set is presented in the respective figure legend. Adjusted *p* < 0.05 was considered to indicate a statistically significant difference.

## Data Availability

Data is contained within the article or Supplementary Material. The data presented in this study are available within the article and in Supplementary Figure S1a,b.

## Supplementary Materials

The following are available online at https://www.mdpi.com/1422-0067/22/1/298/s1, Figure S1a: Immunohistochemistry was used to confirm the RPE phenotype in the cultured AR-PE-19 cell line using antibodies against retinal pigment epithelium-65 (RPE-65). Figure S1b: To confirm that the DMSO solution used to dissolve the tonabersat did not produce cytotoxic effects, a DMSO treatment group (HG + Cyt + DMSO) was added.
