# Supplementary material for: Tonabersat Inhibits Connexin43 Hemichannel Opening and Inflammasome Activation in an In Vitro Retinal Epithelial Cell Model of Diabetic Retinopathy
Source: Int J Mol Sci. 2020 Dec 30;22(1):298. doi: 10.3390/ijms22010298 (PMC7794685; doi:10.3390/ijms22010298)
Supplement: Supplementary file 1 [file ijms-22-00298-s001.pdf]

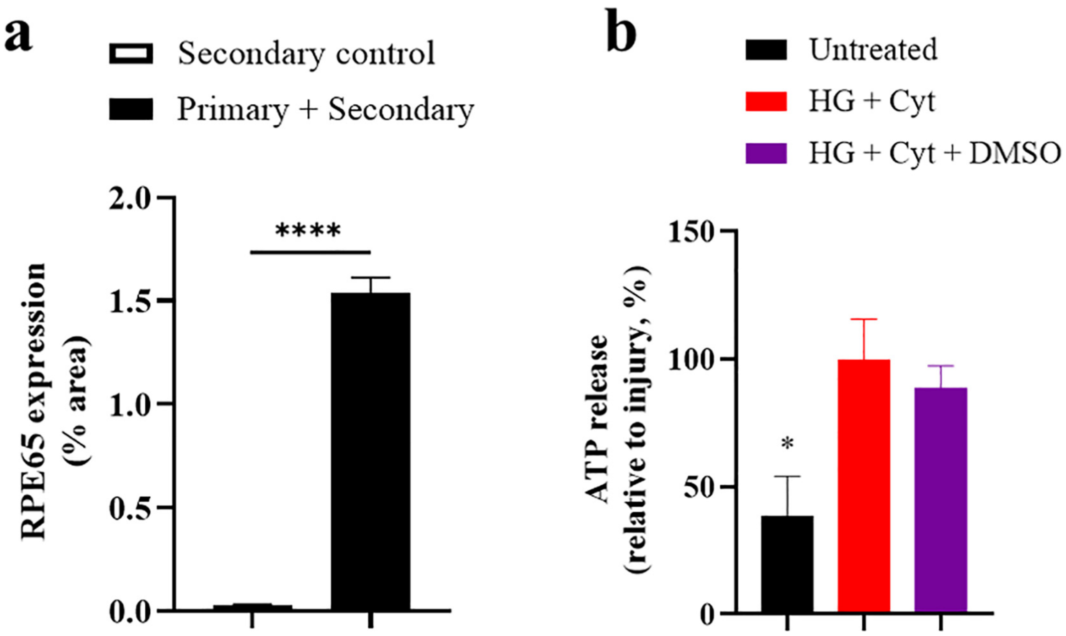

Figure S1: (a): Immunohistochemistry was used to confirm the RPE phenotype in the cultured ARPE-19 cell line using antibodies against retinal pigment epithelium-65 (RPE-65). (b): To confirm that the DMSO solution used to dissolve the tonabersat did not produce cytotoxic ef-fects, a DMSO treatment group (HG + Cyt + DMSO) was added. * $p < 0.05$; **** $p \leq 0.0001$
